# Associations among Human-Associated Fecal Contamination, *Microcystis aeruginosa*, and Microcystin at Lake Erie Beaches

**DOI:** 10.3390/ijerph120911466

**Published:** 2015-09-11

**Authors:** Cheonghoon Lee, Jason W. Marion, Melissa Cheung, Chang Soo Lee, Jiyoung Lee

**Affiliations:** 1 Division of Environmental Health Sciences, College of Public Health, Ohio State University, Columbus, OH 43210, USA; E-Mails: shota2@snu.ac.kr (C.L.); jason.marion@eku.edu (J.W.M.); cheung.mya@gmail.com (M.C.); changsoo@kribb.re.kr (C.S.L.); 2 Department of Food Sciences & Technology, Ohio State University, Columbus, OH 43210, USA

**Keywords:** harmful algal bloom, cyanotoxin, microbial source tracking, urban beaches, Lake Erie

## Abstract

Lake Erie beaches exhibit impaired water quality due to fecal contamination and cyanobacterial blooms, though few studies address potential relationships between these two public health hazards. Using quantitative polymerase chain reaction (qPCR), *Microcystis aeruginosa* was monitored in conjunction with a human-associated fecal marker (*Bacteroides fragilis* group; g-Bfra), microcystin, and water quality parameters at two beaches to evaluate their potential associations. During the summer of 2010, water samples were collected 32 times from both Euclid and Villa Angela beaches. The phycocyanin intergenic spacer (PC-IGS) and the microcystin-producing (mcyA) gene in *M. aeruginosa* were quantified with qPCR. PC-IGS and mcyA were detected in 50.0% and 39.1% of samples, respectively, and showed increased occurrences after mid-August. Correlation and regression analyses showed that water temperature was negatively correlated with *M. aeruginosa* markers and microcystin. The densities of mcyA and the g-Bfra were predicted by nitrate, implicating fecal contamination as contributing to the growth of *M. aeruginosa* by nitrate loading. Microcystin was correlated with mcyA (*r* = 0.413, *p* < 0.01), suggesting toxin-producing *M. aeruginosa* populations may significantly contribute to microcystin production. Additionally, microcystin was correlated with total phosphorus (*r* = 0.628, *p* < 0.001), which was higher at Euclid (*p* < 0.05), possibly contributing to higher microcystin concentrations at Euclid.

## 1. Introduction

Lake Erie, which is one of the five Laurentian Great Lakes, provides water for drinking and recreation for 11 million people [1]. Water recreation is one of the most popular outdoor activities in the United States [2] and along the Ohio shoreline of Lake Erie there are 63 public and semi-public beaches for water recreational activities [3]. However, these coastal Ohio beaches located on Lake Erie were ranked worst in the nation for the frequency of beach advisories based on *E. coli* levels (30th out of 30 coastal states) [3], which suggests a risk of gastrointestinal disease for Ohio’s beachgoers. 

Euclid and Villa Angela beaches are part of Cleveland Lakefront State Park, which is among the most visited parks in Ohio (2010 total visitor occasions: 9,285,452) [4]. However, these two urban beaches are highly contaminated as exhibited by these beaches exceeding the daily maximum *E. coli* standard in 44% and 40% of beach water samples during the 2010 swimming season [5]. The beach water at these locations is contaminated with point and nonpoint sources originating from humans and wildlife, such as gulls [6,7], and loadings of human pathogens and nutrients associated with fecal contamination may be higher at these beaches than other Lake Erie beaches.

In addition to infectious disease risk from potential enteric pathogens, harmful algal blooms have frequently been observed, especially in western Lake Erie, and *Microcystis* has dominated the cyanobacterial bloom community [8,9,10,11]. More specifically, *Microcystis aeruginosa*, which is one of the most dominant species within the *Microcystis* group, has been associated with the massive algal blooms of the last decade [10,12]. This species can produce hepatotoxic microcystin, which is a public health concern because it can cause acute and chronic diseases in human and animals [13]. For recreational waters, the World Health Organization has developed guideline values at three levels [14]: potential human health risk in recreational waters is considered relatively low at 20,000 cyanobacterial cells·mL^−1^ (10 μg·L^−1^ chlorophyll *a*), moderate at 100,000 cyanobacterial cells·mL^−1^ (50 μg·L^−1^ chlorophyll *a*), and high over 100,000 cells·mL^−1^. At 20,000 cyanobacterial cells·mL^−1^, 20 μg·L^−1^ of microcystin is likely if the bloom consists of *Microcystis* and has an average toxin content of 0.2 pg per cell, or 0.4 μg microcystin per 1 μg chlorophyll *a* [14]. The Ohio Environmental Protection Agency monitors the concentrations of microcystin and other toxins from Lake Erie and inland beach waters and has two advisory levels [15]: recreational public health advisory (6 μg·L^−1^ of microcystin) and no contact advisory (20 μg·L^−1^ of microcystin). In 2011, it was reported that the microcystin concentration at Euclid Beach (6.1 μg·L^−1^) once exceeded the recreational public health advisory level [15]. 

It has been well known that nutrient loading contributes to the production of microcystin by promoting the growth of toxin-producing *M. aeruginosa* [16,17,18] in Lake Erie. Human fecal contamination at Lake Erie urban beaches may be associated with nutrient loading, which may promote microcystin production. *Bacteroides* spp. are abundant in human feces [19] and are regarded as a better indicator of human-associated fecal contamination than other fecal markers [20,21] and a qPCR assay targeting the 16S rRNA gene of the *B. fragilis* group (g-Bfra) [22] could provide information on the extent of human-associated fecal contamination. Moreover, microcystin concentrations may be different at neighboring beaches due to different distributions of nutrients. The microcystin concentrations in higher nutrient-containing bathing waters may be higher than at neighboring bathing areas with lesser nutrients. In the current study, total and toxin-producing *M. aeruginosa* in two neighboring Lake Erie urban beaches were monitored using qPCR assays targeting the phycocyanin intergenic spacer (PC-IGS) [23] and microcystin synthetase gene *mcyA* (mcyA) [18]. This monitoring occurred in conjunction with the quantification of a human-associated fecal marker (g-Bfra) [22], microcystin, and nutrients to determine if levels of human-associated fecal contamination and microcystin are positively associated with each other at these two urban Lake Erie beaches. 

## 2. Experimental Section 

### 2.1. Site Description and Water Sampling Collection

**Figure 1:**
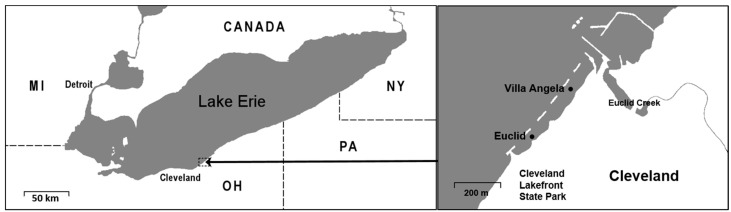
Two beach sites (sampling sites) in Lake Erie. Euclid (41°35′9″N; 81°34′1″W); Villa Angela (41°35′2″N; 81°34′9″W).

This study was conducted at Euclid (41°35′9″ N; 81°34′1″ W) and Villa Angela, two beaches (41°35′2″ N; 81°34′9″ W) near Cleveland, Ohio (Figure 1). The 200-m Euclid Beach adjoins the 300-m Villa Angela Beach, which extends to a dividing pier that separates the beach from the mouth of Euclid Creek. Detached breakwaters constructed out of armor stone are located at both beaches. These breakwaters exist to reduce shoreline erosion and permit appropriate sand drift and placement through reduced wave energy and action. Although the detached design ideally permits shore-perpendicular movement of water and sand, this design can reduce water circulation from outer areas, which may lead to entrainment of nutrients and potential localized enrichment. Elevated nutrient and bacterial loadings into this beach environment have occurred previously through storm sewer outfalls located along Euclid Creek. These outfalls have been implicated in providing human-associated fecal bacteria contamination to the study beaches through combined sewer overflows (CSOs) and sanitary sewer overflows (SSOs) [24,25]. Shorebirds such as gulls and geese may also contribute to the nutrient and bacterial loading at these two beaches [7]. 

Using the Ohio Department of Health sampling guidelines for beach waters [5], beach water samples (>2 L) were collected 32 times in 750 mL whirl-pak^®^ bags (Nasco, Fort Atkinson, WI, USA) from each of the two beaches from 15 July to 15 September 2010. After collection, samples were transported to a nearby temporary field laboratory at the Ohio State University Extension office in Painesville (Lake County, OH) on ice, and filtered within an hour. Beach water samples (200 mL) were filtered through a 47-mm-diameter, 0.45-μm-pore-sized membrane (Pall Corporation, Ann Arbor, MI, USA). Membrane filters were transferred into a 50-mL sterile tube. Water samples and the membrane filters were sent to the Ohio State University main campus laboratory (Columbus, OH) on ice for further analysis. Precipitation levels were obtained from the National Oceanic and Atmospheric Administration [26] for the day prior and day of sample collection using data from Cleveland Burke Lakefront Airport (Station ID: GHCND:USW00004853), located 12.5 km west of the study site. Water temperature, pH, and conductivity were measured *in situ* using a multiparameter water quality sonde (YSI 600XL; Yellow Springs Instruments, Yellow Springs, OH, USA). Turbidity was measured with a Hach Turbidimeter (Hach Company, Loveland, CO, USA). Phycocyanin and chlorophyll *a* were quantified *in vivo* using a two-channel handheld AquaFluor™ fluorometer (Turner Designs^®^, Sunnyvale, CA, USA) as described in our previous study [27]. Chlorophyll *a* was standardized (*R^2^* = 99.9%) with liquid primary chlorophyll *a* standards (Turner Designs, Sunnyvale, CA, USA). Phycocyanin was standardized (*R^2^* = 99.9%) using a lyophilized powder containing approximately 30% c-phycocyanin from *Spirulina* sp. (Sigma-Aldrich Co. LLC, St. Louis, MO, USA). The starting mass of the phycocyanin powder used in the stepwise dilution and subsequent standard curve generation was multiplied by 0.3 to reflect the true mass of phycocyanin contained in the powder.

### 2.2. Measurement of Nutrients

Colorimetric methods were used to determine the concentration of nitrate with the cadmium reduction method using Hach Method 8192 (Hach Company, Loveland, CO, USA). Total phosphorus concentrations were determined by PhosVer 3 with acid persulfate digestion using Hach Method 8190 (Hach Company, Loveland, CO, USA). Before the measurement, all glassware was cleaned with 6.0 N (1:1) hydrochloric acid, then rinsed with deionized water to remove contaminants. Both measurements were carried out according to the standard methods [28]. For the standard curves, nitrate in the range of 0-500 μg·L^−1^ and total phosphorus in the range of 0–30 μg·L^−1^ were prepared by serially diluting 1.6 mM NaNO_3_ and 0.3 mM K_2_HPO_4_ (Sigma-Aldrich Co. LLC, St. Louis, MO, USA) solutions using pure water. All measurements were carried out in triplicate. Accuracies of the measurements were over 99% in the ranges of nitrate and total phosphorus by comparing theoretical and experimental nutrient concentrations measured by Hach methods. Potential interference of freshwater matrix was tested with the serially-diluted standard solutions using natural freshwater inhabited by *M. aeruginosa* as described above. The accuracies of nitrate and total phosphorus measurements were 97% and 96%, respectively, suggesting that the interference of freshwater matrix was negligible.

### 2.3. Measurement of Microcystin

Microcystin concentrations from beach water samples were quantified using the U.S. Environmental Protection Agency-validated Microcystins/Nodularins ([2*S*,3*S*,8*S*,9*S*]-3-Amino-9-methoxy-2,6,8-trimethyl-10-phenyldeca-4,6-dienoic acid [ADDA]) ES, ELISA kit in a 96-well format (catalog number, PN520011ES; Abraxis^®^, Warminster, PA, USA) as described previously [27]. Briefly, water samples were subjected to three freeze-thaw cycles to effectively rupture cells and release the toxins [29]. For each multi-well plate, a total of six standards (0 μg·L^−1^, 0.15 μg·L^−1^, 0.4 μg·L^−1^, 1 μg·L^−1^, 2 μg·L^−1^, and 5 μg·L^−1^) were used in duplicate for developing the plate-specific standard curves (mean *R^2^* = 0.993, *R^2^* Range = 0.990–0.997). All samples were quantified in duplicate using a MRX-TC Revelation microplate reader (Dynex Technologies, Inc., Chantilly VA, USA). The assay detects and quantifies numerous variants of currently known microcystins providing a concentration for total microcystins in microcystin-LR equivalents (μg L^−1^ MC-LR eq). There was good agreement between the two measured optical densities for each sample (Pearson correlation = 0.848, *p <* 0.001). Samples between 0.10 and 0.15 μg L^−1^ MC-LR eq were qualified as “detected but not quantified” (DNQ) and samples below 0.10 μg L^−1^ MC-LR eq were considered “non-detects” (NDs) according to the manufacturer’s instruction. 

### 2.4. DNA Extraction

For PC-IGS and mcyA detection and quantification, DNA from a *M. aeruginosa* NIES-843 culture (as a positive control; provided by Dr. Steven Wilhelm, University of Tennessee) and from the beach water filter membranes was extracted using the xanthogenate-sodium dodecyl sulfate (XS) DNA extraction protocol [30] with modification [27]. Briefly, a 100-μL aliquot of *M. aeruginosa* NIES-843 culture was centrifuged at 12,000 rpm for 1 min and the pellet was resuspended into 1 mL of XS buffer. For beach water samples, each filter membrane was transferred into a 2-mL microcentrifuge tube containing 1 mL of XS buffer. They were incubated at 70 °C for 2 h for releasing DNA. After incubation, each sample was vortexed for 10 s, put on ice for 30 min, and centrifuged at 16,000 × *g* for 15 min at 4 °C. The supernatant was transferred to a new tube and mixed with an equal volume of 100% isopropanol. The sample was transferred to a column in the DNeasy^®^ Blood & Tissue Kit (Qiagen, Valencia, CA, USA) and centrifuged for 1 min at 10,000 × *g*. The column was washed with 500 μL of buffer AW1 from the DNeasy^®^ Blood & Tissue Kit, and centrifuged for 1 min at 10,000 × *g*. The column was then washed with 500 μL of buffer AW2 from the DNeasy^®^ Blood & Tissue Kit, and centrifuged for 3 min at 16,000 × *g*. One hundred microliters of AE buffer was added to the column, which was incubated for 1 min. To elute DNA, the column was centrifuged for 1 min at 16,000 × *g*. 

For g-Bfra, DNA from the culture of *B. fragilis* ATCC 25285^T^ (as a positive control; 100 μL) and membranes were extracted as described previously [6]. They were finally suspended in 200 μL of elution buffer. The eluates were used immediately or stored at –20°C until further processing.

### 2.5. Real-Time qPCR

Real-time qPCR was carried out in duplicate with a StepOne^TM^ Real-Time System (Applied Biosystems, Foster City, CA, USA) in a 48-well format with a total reaction mixture volume of 20 μL containing DNA template (2 μL for PC-IGS and mcyA; 5 μL of DNA for g-Bfra), forward and reverse primers (PC-IGS: 500 nM each; mcyA: 200 nM each; g-Bfra: 500 nM each) (Table 1), and 10 μL of SYBR^®^ universal PCR master mix (Applied Biosystems, Foster City, CA, USA). Thermal cycling for PC-IGS and mcyA consisted of an initial cycle of 95 °C for 10 min, followed by 40 cycles of denaturation at 95 °C for 30 s, and annealing and extension at 62 °C for 3 min. A melting curve analysis was performed after amplification to distinguish the targeted PCR product from the false-positive PCR product. The melting curve analysis was performed by heating samples to 95 °C for 30 s, cooling to 62 °C for 3 min, and then heating the samples at 0.3 °C·s^−1^ to 95 °C. Thermal cycling for g-Bfra included an initial cycle of 95 °C for 10 min, followed by 40 cycles of denaturation at 95 °C for 15 s, annealing at 50 °C for 30 s, and extension at 60 °C for 4 min. Cycle threshold (Ct) values of a real-time PCR result were determined from a fixed threshold (0.2 for PC-IGS and mcyA and 0.05 for g-Bfra). DNA from *M. aeruginosa* NIES-843 culture (for PC-IGS and mcyA) and *B. fragilis* ATCC 25285^T^ (for g-Bfra) was used as positive controls. Distilled water was used as a negative control in all assays. Samples showing Ct values in both replicates were regarded as positive detects and the quantification of PC-IGS and mcyA in these positive samples relied on standard curves, which were made as described previously [27] with modifications. Briefly, to generate the standard curve, the 10-fold serial dilutions of plasmids, ranging from 5.7 × 10^7^ to 5.7 × 10^−1^ copies (gene equivalents [GE]) for PC-IGS and from 4.4 × 10^7^ to 4.4 × 10^−1^ GE for mcyA were used. Real-time qPCR assays for standard curve generation were independently performed twice in triplicate. For the quantification of the g-Bfra, 10-fold serial dilutions of *B. fragilis* ATCC 25285^T^ were prepared and followed by a g-Bfra qPCR assay to make a standard curve [31]. The g-Bfra qPCR assay for standard curve generation was independently performed once in triplicate. The linear range of quantification for PC-IGS, mcyA, and g-Bfra was from 5.7 × 10^7^ to 5.7 × 10° GE·reaction^−1^, from 4.4× 10^7^ to 4.4 × 10° GE·reaction^−1^ and from 2.3 × 10^5^ to 2.3 × 10^1^ GE·reaction^−1^, respectively. Standard curves were created by plotting Ct values *versus* the log number of GE within the linear range of quantification and generating a trend line through these points. The slopes and the y-intercepts of the standard curves were –3.3107 and 34.833 for PC-IGS, –3.5853 and 35.867 for mcyA, and –3.5965 and 39.547 for g-Bfra, respectively. Good linear correlations were shown between the GE and Ct values (*R^2^* = 0.9840 for PC-IGS, 0.9955 for mcyA, 0.9898 for g-Bfra). The limit of quantification (LOQ) was based on the lowest range of standards that contributed to the linear part of the standard curve [32]. Therefore, the LOQs for PC-IGS, mcyA, and g-Bfra were determined as 5.7 × 10° GE·reaction^−1^, 4.4 × 10° GE·reaction^−1^, and 2.3 × 10^1^ GE·reaction^−1^, respectively. Negative controls were used to measure a limit of detection (LOD). The negative controls sometimes showed nonspecific fluorescent signal during late cycles. In those cases, the average and the 99-percent confidence interval among cycle thresholds were calculated. If Ct values were undetermined in negative controls, the values were set to the end cycle of thermal cycling (40). To guard against false-positive results, the target concentration that corresponded with detection at the lower 99-percent confidence interval of multiple detections was used as the LOD [32]. A cycle threshold higher than the LOD was considered ND. Results between the LOQ and the LOD were qualified as DNQ.

**Table 1:** Primers used in this study.

Assay Name	Primer	Sequence (5′-3′)	Amplicon Size (bp)	Gene	Target	Reference
PC-IGS	188F	GCTACTTCGACCGCGCC	67	phycocyanin intergenic spacer	*Microcystis aeruginosa*	[23]
	254R	TCCTACGGTTTAATTGAGACTAGCC				
mcyA	M1rF	AGCGGTAGTCATTGCATCGG	107	*mcyA* gene		[18]
	M1rR	GCCCTTTTTCTGAAGTCGCC				
g-Bfra	g-Bfra-F	ATAGCCTTTCGAAAGRAAGAT	501	16S rRNA gene	*Bacteroides fragilis*	[22]
	g-Bfra-R	CCAGTATCAACTGCAATTTTA				

### 2.6. Determination of PCR Inhibition in Beach Water Samples

To determine the possible presence of PCR inhibition in water samples, the Sketa22 qPCR assay [30] modified from Haugland *et al.* [33] and U.S. Environmental Protection Agency [34] was performed in duplicate with a StepOne^TM^ Real-Time System (Applied Biosystems, Foster City, CA, USA). For PC-IGS and mcyA, the qPCR mixture consisted of a total volume of 25 μL containing 5 μL of the DNA extract, 1 μL of the diluted salmon DNA solution (10 ng·μL^−1^; Invitrogen, Carlsbad, CA, USA), 12.5 μL of TaqMan^®^ Universal PCR Master Mix (Applied Biosystems, Foster City, CA, USA), 2.5 μL of bovine serum albumin (2 mg mL^−1^, Sigma-Aldrich, St. Louis, MO, USA), 5 μM of each forward (SketaF2: 5′-GGTTTCCGCAGCTGGG-3′) and reverse (SketaR3: 5′-CCGAGCCGTCCTGGTCTA-3′) primer, and 400 nM of probe (SketaP2: 5′-FAM/ AGTCGCAGGCGGCCACCGT/MGB-3′) [33]. The PCR protocol included an initial cycle at 50 °C for 2 min and 95 °C for 10 min, followed by 45 cycles of denaturation at 95 °C for 15 s, annealing and extension at 60 °C for 1 min [33]. Ct values of the qPCR result were determined from the threshold 0.2. PCR inhibition was determined as described previously [7]. To determine the presence of natural salmon sperm DNA in the beach water samples, the Sketa22 qPCR assay was performed with the DNA template of each sample without adding salmon sperm DNA. All of the DNA extracts for g-Bfra did not show any significant PCR inhibition, as determined previously [7].

### 2.7. Statistical Analysis

All statistical analyses were performed with the IBM^®^ SPSS^®^ (Release ver. 19.0.0; SPSS Inc., Chicago, IL, USA). Before analysis, non-normally distributed data, according to the Shapiro-Wilk test, were log transformed. A paired-sample t test was used for comparing the values of parameters between the two sampling sites and one-way analysis of variance (ANOVA) was used for comparing the parameter values between the sampling periods at both beaches. Spearman correlation analysis was used for testing the possible correlations among g-Bfra, PC-IGS, mcyA, microcystin, and environmental parameters. Correlation analysis was followed by multiple linear regression analysis using backward selection (Probability-of-F-to-enter ≤ 0.050, Probability-of-F-to-remove ≥ 0.100), which was used to assess the relationships between qPCR markers, microcystin, and water quality variables. For addressing colinearity, variance inflation factors (VIF) were evaluated in the multiple regression analysis, and water quality variables showing colinearity (VIF > 10) were removed individually during the construction of the most parsimonious models. Using the backward selection procedure, variables with *p* < 0.15 were considered for the model [35]. Chlorophyll *a*, phycocyanin, PC-IGS, and mcyA were not included in this analysis as they were assumed to be partially correlated with water quality parameters (predictor variables) as the response variables [36,37]. For log-transformation and mean calculation, DNQs of genetic markers were assigned a value of half the limit of quantification (LOQ/2) for genetic markers and 0.13 μg·L^−1^ for microcystin, and NDs of genetic markers were treated as 1 GE·L^−1^ for genetic markers and 0.05 μg·L^−1^ for microcystin.

## 3. Results

### 3.1. Water Quality Parameters

**Figure 2:**
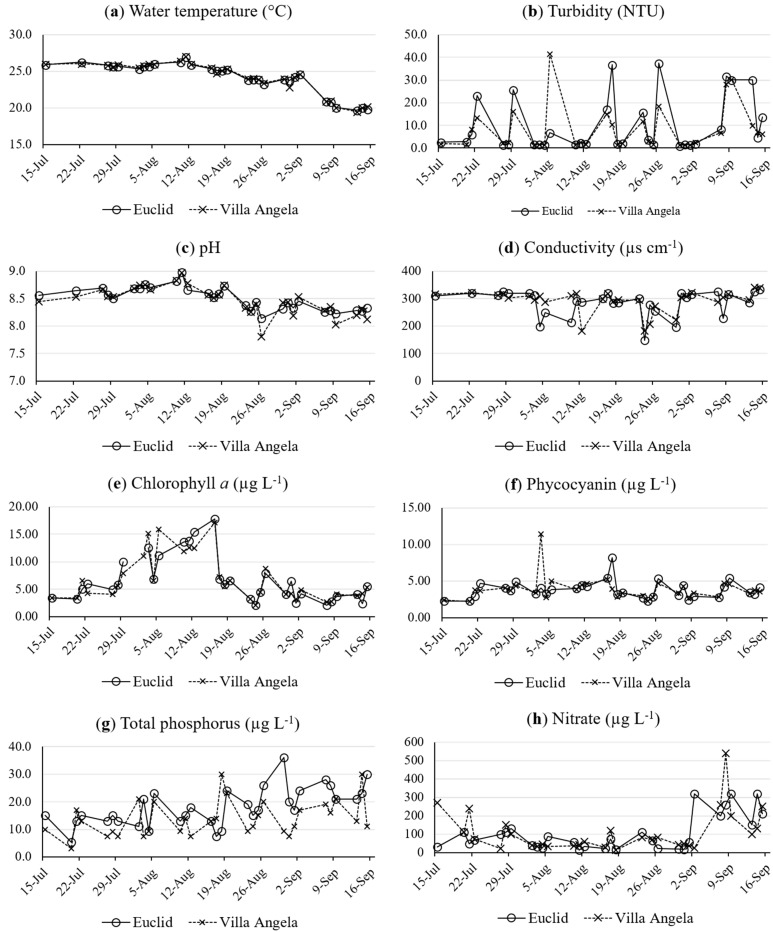
Profiles of water quality parameters at Euclid and Villa Angela beaches from July to September in 2010: (**a**) water temperature; (**b**) turbidity; (**c**) pH; (**d**) conductivity; (**e**) chlorophyll *a*; (**f**) Phycocyanin; (**g**) total phosphorus; (**h**) nitrate.

The results of the water quality parameters at both beaches are presented in Figure 2. Mean and median values for most water quality parameters were similar among the sampling sites. Only the mean values of total phosphorus were significantly different between the two beach sites (Euclid: 18 ± 6.9 [mean ± standard deviation] μg·L^−1^; Villa Angela: 14 ± 6.5 μg·L^−1^) according to the paired-sample t-test (*p* = 0.004). At both beaches, water temperature, pH, and chlorophyll *a* peaked in early August (Figure 2a,c,e), whereas the concentrations of total phosphorus and nitrate were higher in late August and September, respectively (Figure 2g,h). Measurable precipitation events (>0.0254 cm) were observed on six sampling dates, with < 0.4 cm being observed on 22 and 28 July, 3 and 4 August, and 7 September. On 11 August, we recorded the maximum sample day precipitation of 1.4 cm. With respect to rainfall one day prior to sample collection, measurable previous day rainfall was observed for five sample dates, with < 0.4 cm being recorded for 29 July, 4 and 5 August, and 8 September. The maximum previous day rainfall total recorded was 2.5 cm for 15 July. Total phosphorus showed negative correlation with water temperature (*r* = −0.380) and pH (*r* = −0.316), while nitrate was correlated with water temperature (*r* = −0.426), turbidity (*r* = 0.416), pH (*r* = −0.496), conductivity (*r* = 0.336), and chlorophyll *a* (*r* = –0.448) according to Spearman correlation analysis (*p* < 0.05). Previous day rainfall was not associated with water quality terms, but same-day rainfall was positively correlated with water temperature (*r* = 0.431), pH (*r* = 0.378), and chlorophyll *a* (*r* = 0.293). No associations between rainfall and nutrients were observed. Lastly, the mean concentrations of phosphorus and chlorophyll *a* (Figure 2e) indicate that the water at both beaches was in mesotrophic status (Carlson Index) during the study period [38].

### 3.2. qPCR Results

PC-IGS and mcyA were frequently detected from a total of 64 beach water samples (32 out of 64 [50.0%] for PC-IGS and 25 out of 64 [39.1%] for mcyA). The occurrence of the PC-IGS marker was higher at Villa Angela, whereas mcyA occurrence was similar at both beaches (Table 2). The densities of PC-IGS and mcyA ranged from ND (qPCR-negative) to 3.1 × 10^5^ GE·L^−1^ and from ND to 1.6 × 10^5^ GE·L^−1^, respectively. The mean values calculated from all the samples for both markers were slightly higher at Villa Angela (Table 2); however, the mean values were not statistically different according to the paired-sample t-test (*p* > 0.05). g-Bfra was also frequently detected from beach water samples (32 out of 58 [55.2%]). The g-Bfra occurrence at Villa Angela and Euclid were similar (Table 2). The g-Bfra densities ranged from ND (qPCR-negative) to 3.3 × 10^5^ GE L^−1^ and the mean values of all samples and qPCR-positive samples for g-Bfra were higher at Villa Angela (Table 2), although their differences were not statistically significant according to the paired-sample t-test (*p* > 0.05). 

**Table 2:** Occurrence, means, and range of genetic marker densities and microcystin concentrations at Euclid and Villa Angela beaches.

Beach	Statistic	PC-IGS (GE L^−1^)	mcyA (GE L^−1^)	g-Bfra (GE·L^−1^)	Microcystin (μg L^−1^ MC-LR eq)
Euclid	Occurrence ^a^	13/32 (40.6)	12/32 (37.5)	16/29 (55.2)	13/31 (41.9)
	Mean ± SD ^b^ (range)	1.7 × 10^4^ ± 2.5 × 10^4^ (ND-1.8 × 10^5 c^)	5.6 × 10^3^ ± 7.7 × 10^3^ (ND-5.9 × 10^4^)	2.1 × 10^4^ ± 4.0 × 10^4^ (ND-1.9 × 10^5^)	0.11 ± 0.10 (ND-0.47)
Villa Angela	Occurrence	19/32 (59.4)	13/32 (40.6)	16/29 (55.2)	6/32 (18.8)
	Mean ± SD (range)	3.9 × 10^4^ ± 7.5 × 10^4^ (ND-3.1 × 10^5^)	1.3 × 10^4^ ± 2.9 × 10^4^ (ND-1.6 × 10^5^)	3.0 × 10^4^ ± 6.9 × 10^4^ (ND-3.3 × 10^5^)	0.07 ± 0.053 (ND-0.28)

^a^ Number of positive samples/total samples (%); ^b^ Arithmetic mean ± standard deviation. ^c^ ND: not detected.

**Figure 3:**
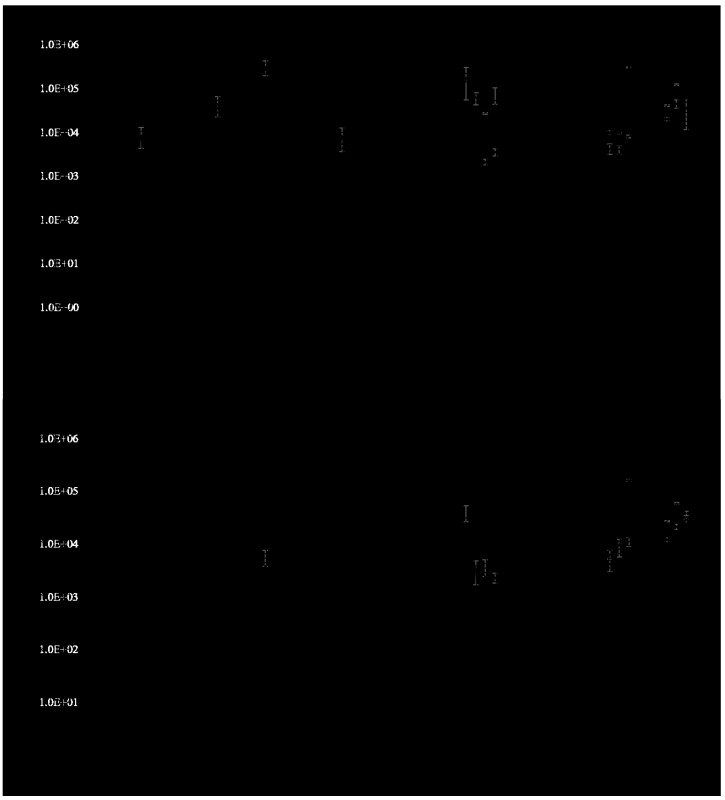

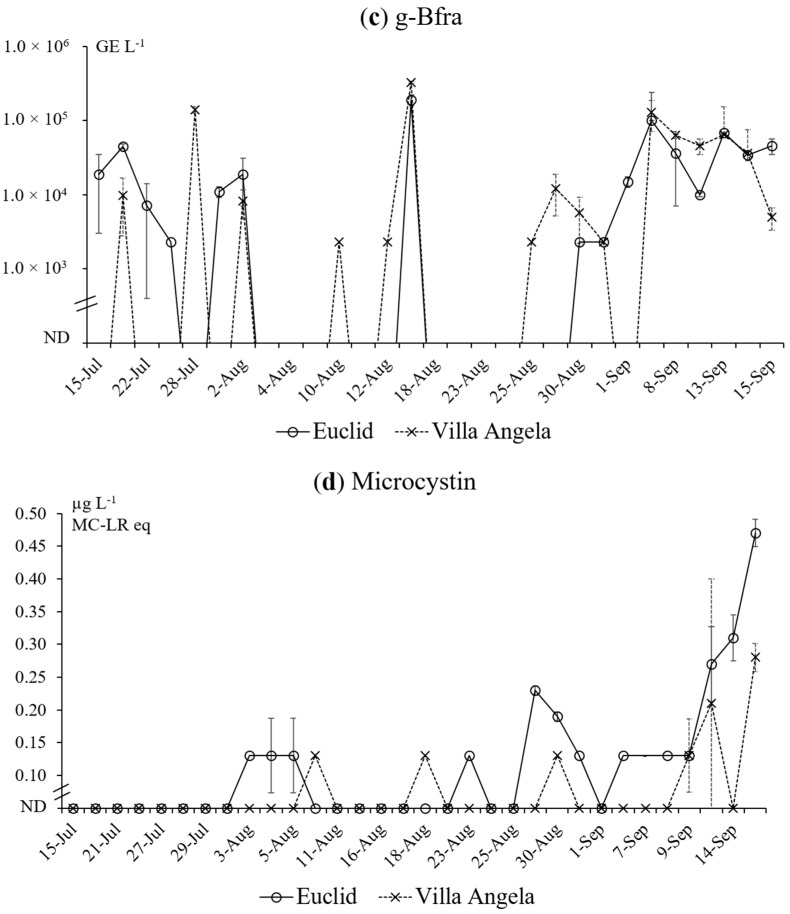
Profiles of the genetic marker densities and microcystin concentrations at Euclid and Villa Angela beaches from July to September in 2010. The error bars represent standard deviations (Euclid: solid lines, Villa Angela: dashed lines). The negative values of standard deviations higher than mean values are not included. g-Bfra densities in the water samples taken on 20 July, 16 August, and 2 September were not measured. ND: not detected. (**a**) PC-IGS; (**b**) mcyA; (**c**) g-Bfra; (**d**) microcystin.

Overall, each qPCR marker was frequently detected in late August to September at both beaches (Figure 3a–c). PC-IGS and g-Bfra markers were also frequently detected from late July to early August at Villa Angela (Figure 3a) and late July at both beaches (Figure 3c), respectively. The mcyA densities were higher in September at both beaches compared to August and July according to the ANOVA (*p* < 0.05) (Figure 3b). 

Melting curve analysis showed that no primer dimers were formed from all the qPCR-positive samples, and the melting temperature (Tm) values of all the qPCR product peaks ranged from 82.3 to 83.1 °C for PC-IGS, from 79.2 to 79.6 °C for mcyA, and from 79.2 to 79.6 °C for g-Bfra, which were closer to the mean Tm values of PC-IGS (83.1 °C), mcyA (79.5 °C), and g-Bfra (83.2 °C) positive controls, respectively. 

The Sketa22 qPCR assay was used to determine whether inhibition was present in DNA extracts for PC-IGS and mcyA qPCR assays ,as PCR inhibition can generate false negatives and/or biases in the qPCR results. By comparing the ΔCt value from each test sample (Ct value_test sample_ − mean Ct value_controls_), no significant inhibition was shown in these samples (ΔCt < 1.5). No positive results were observed from the Sketa22 assay when salmon sperm DNA was not spiked. 

### 3.3. Microcystin Measurement

Microcystin was detected in 19 out of the 63 total (30.2%) beach water samples. The occurrence of microcystin-positive samples was nearly two times higher at Euclid than Villa Angela (Table 2); and overall, microcystin concentrations ranged from <0.1 (below detection limit) to 0.47 μg·L^−1^ MC-LR eq (Table 2). The mean values of microcystin concentrations were higher at Euclid (Table 2) and the distribution of microcystin was statistically different according to the paired-sample t-test (*p* = 0.007). Microcystin was detected from early August at both beaches (Figure 3d) and frequently detected from late August at Euclid and early September at Villa Angela (Figure 3d). The microcystin concentrations at both beaches increased in September and reached peak concentrations in late September (Figure 3d). 

### 3.4. Relationships among QPCR Results, Microcystin, and Water Quality Parameters

Spearman correlation analysis showed several significant relationships among qPCR results, microcystin, and water quality parameters (Table 3). PC-IGS and mcyA were strongly correlated with each other (*r* = 716, *p* < 0.001) and with g-Bfra, microcystin, most water quality parameters, and nitrate (*p* < 0.01). Among water quality parameters, water temperature had the strongest correlation with both genetic markers (Table 3). g-Bfra was correlated with *M. aeruginosa* markers, most water quality parameters, chlorophyll *a*, and nitrate. Among all parameters, mcyA, water temperature, and nitrate had the strongest correlations with g-Bfra (Table 3). Microcystin was positively correlated with *M. aeruginosa* markers and total phosphorus and negatively correlated with water temperature and pH (Table 3). Among the qPCR markers, mcyA was significantly correlated with the qPCR markers (Table 3). Multiple regression analyses for PC-IGS, mcyA, and g-Bfra show that water temperature and nitrate were common variables for predicting the densities of mcyA and g-Bfra densities. Water temperature was the only variable for predicting PC-IGS densities. With respect to microcystin concentrations, both water temperature and total phosphorus were significant predictors (*p* < 0.01) (Table 4). Based on adjusted *R^2^* values, regression models for PC-IGS, mcyA, and microcystin were relatively strong (adjusted *R^2^* > 0.500), whereas the g-Bfra model was relatively weaker (adjusted *R^2^* = 0.336).

**Table 3:** Summary of Spearman correlation coefficient showing significant relationships among qPCR measurements, microcystin, and water quality parameters.

Variable	PC-IGS	mcyA	g-Bfra	Microcystin
PC-IGS	−	0.716 ***	0.411 **	0.414 **
mcyA	0.716 ***	−	0.535 ***	0.413 **
g-Bfra	0.411 **	0.535 ***	−	NC
Microcystin	0.414 **	0.413 **	NC	−
Water temperature	−0.701 ***	−0.750 ***	−0.542 ***	−0.470 ***
Turbidity	NC	0.384 **	0.341 **	NC
pH	−0.632 ***	−0.673 ***	−0.431 **	−0.369 **
Conductivity	NC	NC	0.363 **	NC
Chlorophyll *a*	−0.386 **	−0.407 **	−0.325 *	NC
Phycocyanin	NC	NC	NC	NC
Total phosphorus	NC	0.336 **	NC	0.628 ***
Nitrate	0.462 ***	0.586 ***	0.547 ***	NC

* *p* < 0.05; ** *p* < 0.01; *** *p* < 0.001. NC: not correlated.

**Table 4:** Parameters of multiple linear regression models explaining genetic marker densities (log) and microcystin concentrations (log) from beach water samples.

Dependent Variable	Water Quality Explanatory Variable	Regression Coefficient (β)	Standard Error of β	Standardized Regression Coefficient (β)	*t* Test Value	Sig. (*p*)	*R^2^*	Adjusted *R^2^*	Model Sig.
PC-IGS (log)	Constant	50.743	6.387		7.945	<0.001	0.519	0.511	<0.001
	Water temperature (log)	−35.320	4.623	−0.721	−7.640	<0.001			
		
mcyA (log)	Constant	43.662	6.486		6.731	<0.001	0.683	0.671	<0.001
	Water temperature (log)	−31.649	4.318	−0.708	−7.330	<0.001			
	Nitrate (log)	0.825	0.448	0.178	1.843	0.071			
g-Bfra (log)	Constant	29.747	10.839		2.745	0.008	0.336	0.322	<0.001
	Water temperature (log)	−21.505	7.166	−0.433	−3.001	0.004			
	Nitrate (log)	1.287	0.784	0.237	1.642	0.107			
Microcystin (log)	Constant	2.028	0.909		2.230	0.003	0.554	0.538	<0.001
	Water temperature (log)	−2.520	0.637	−0.388	−3.957	<0.001			
	Total phosphorus	0.020	0.004	0.494	5.035	<0.001			

## 4. Discussion 

In this study, we investigated the occurrence of *M. aeruginosa* genetic markers (total and toxin-producing), microcystin, and a genetic marker for human-associated fecal contamination at two Lake Erie beaches. It has been reported that these beaches are frequently contaminated with fecal pollution [5]. Human fecal inputs likely occur frequently at these urban beaches since g-Bfra was detected from more than 50% of beach water samples in this study (Table 2) and other human-specific fecal markers were also frequently detected at these beaches in previous studies [6,7]. In addition, nontoxic and toxin-producing *M. aeruginosa* were also prevalent at these beaches (Table 2).

The finding of no significant correlation between chlorophyll *a* and phycocyanin or microcystin (Table 3) is inconsistent with previous research that demonstrated a positive correlation between these two pigments and microcystin in western Lake Erie; Rinta-Kanto *et al.* reported a strong positive correlation between both pigments and microcystin from western Lake Erie water samples [17]. Some potential reasons for inconsistent findings here may relate to three major study differences. The first difference, nutrient availability, may relate to the locations of the sampling efforts between the studies. The beaches assessed here are located more toward the central Lake Erie basin rather than the western basin (where Maumee Bay is located), which was assessed by Rinta-Kanto *et al.* [17]. The western Lake Erie basin is typically eutrophic, and in the previous research by Rinta-Kanto *et al.*, the levels of phosphorus and chlorophyll *a* observed were indicative of eutrophic to hypereutrophic conditions [17]. The two urban beaches assessed here, Villa Angela and Euclid, presented mesotrophic conditions based upon our total phosphorus concentrations (Figure 2). The second difference between the studies was the amount of microcystin observed. Toxin concentrations in this study never exceeded 0.47 μg·L^−1^ MC-LR eq, whereas, some samples from Rinta-Kanto *et al.* were 14.0 and 21.7 μg·L^−1^ MC-LR eq [17]. A third major difference between the studies pertains to sampling spots. This study was performed in the nearshore/littoral zone environment where bathing activities occur, rather than the pelagic zone studied by Rinta-Kanto *et al.* [17]. Therefore, this study represents a unique environment and perspective (beach water), and although speculative, predicting microcystin concentrations by chlorophyll *a* or phycocyanin in Lake Erie may be applicable only in specific aquatic conditions, such as when nutrient levels and/or toxin concentrations are substantially higher than observed in this study.

Not assessed in other studies is the role of human fecal contamination on cyanobacteria proliferation and toxin production. Here, a positive relationship was observed, and one possible explanation of the positive relationship between *M. aeruginosa* and g-Bfra markers based on the Spearman correlation analysis (Table 3) and the multiple regression analysis (Table 4) in this study is that human-associated fecal contamination may have provided sufficient nitrate loading at the studied urban beaches to explain some of the increase in PC-IGS and mcyA. It has been reported that there are possible relationships between *M. aeruginosa* (total, toxin-producing, and the relative abundance of the toxin-producing population) and nitrogen compounds including nitrate [39,40,41]. Specifically, Yoshida *et al.* reported in their Japanese lake study that high nitrate loading may be a significant factor for promoting the growth of toxin-producing *M. aeruginosa* among the total *M. aeruginosa*, which is in agreement with our results that demonstrated mcyA was positively correlated with nitrate (Table 3) [18]. In attempting to understand the sources of nitrate in these beach environments, the g-Bfra regression model was constructed. Among the models constructed in Table 4, this model explained the least amount of variability compared to the other models, and nitrate was associated at *p =* 0.07 rather than *p <* 0.05. One possible explanation regarding the lower *R^2^* value in this model is that animal-associated fecal contamination fertilizer runoff (e.g. golf courses, lawns, *etc.*) may also contribute to nitrate loading and conductivity at the studied beaches, which could possibly lessen the strength of the association between g-Bfra and PC-IGS, mcyA, and microcystin. We did not find significant associations (*p* < 0.05) between the surrogate for runoff (precipitation) and any of the genetic markers or microcystin. Such associations may exist; however, the study was limited due to dry conditions observed during and immediately prior to sampling. Future study exploring relationships during dry *versus* wet weather is recommended, but an adequate number of both wet and dry weather days would need to be observed for appropriate analysis.

It was shown that water temperature was most strongly and negatively related with PC-IGS and mcyA during the study period according to Spearman correlation and multiple regression analyses (Table 3 and Table 4). This phenomenon is different from another study conducted in Lake Erie that reported no significant correlation between water temperature and total and toxin-producing *M. aeruginosa* [17]. This discrepancy is not surprising because the water temperature from mid-July to September in this study was 25 ± 2°C (Figure 2a), which is near the optimal temperature of *M. aeruginosa* [42]. The correlation between water temperature and *M. aeruginosa* could be merely a statistical association explained by other correlated water quality parameters such as pH, which were also correlated with water temperature and *M. aeruginosa* markers. Te and Gin reported that pH was also positively correlated with *Microcystis mcyE* gene copies [37]. High pH conditions may give an advantage to the growth of cyanobacteria because they can provide cyanobacteria more efficient active transport systems for carbon dioxide and bicarbonate [43]. In addition, nitrate concentrations increased in September, which may contribute to the strong negative correlation between *M. aeruginosa* markers and water temperature. 

Microcystin data show that both the occurrence and the concentrations generally increased from late August until the end of the study period in mid-September (Figure 3d). The positive correlation between mcyA and microcystin (*r* = 0.413, *p* < 0.01) suggests that the toxin-producing *M. aeruginosa* populations may contribute significantly to the microcystin production at these study sites. In addition, similar to the PC-IGS and mcyA results, microcystin concentration was more closely associated with water temperature (Table 3 and Table 4). The negative correlation between microcystin and water temperature could be explained by relationships between *M. aeruginosa* growth and optimal temperatures for toxin production. The water temperature during the sampling period ranged from 20 to 27 °C (Figure 3a), which is close to the optimal temperature (between 20 and 25 °C) for microcystin production by *M. aeruginosa* [44,45,46]. As a result, microcystin could have been increasing in the beach water at the study sites during the sampling period as described above. Weather and current may also drive microcystin accumulation in the studied sites. The scenarios of accumulation and toxin drift are plausible as the typical half-life of microcystin is three to 10 weeks under natural summer conditions, with the longer half-life more probable under the pH conditions observed in this study [47]. In addition, total phosphorus was one of the variables predicting the microcystin concentrations (Table 4) and total phosphorus was correlated with mcyA (Table 3) at the studied beaches, which is consistent with previous studies from Canadian lakes [48], Finnish lakes [36], a Polish reservoir [49], and Lake Erie [17]. The association between microcystin and total phosphorus could explain higher microcystin concentrations at Euclid, as the mean values of total phosphorus were significantly different between Euclid and Villa Angela according to the paired-sample t-test (*p* < 0.05). These results are not surprising in the Laurentian Great Lakes, where phosphorus concentrations have been linked to microcystin levels when nitrate levels were not correlated in Lake Ontario [50]. It is plausible that microcystin concentrations may differ by location at the same beach or at neighboring beaches due to differing distributions of potentially growth- and toxin-limiting nutrients. Consequently, it is possible that people swimming in higher nutrient-containing bathing areas may be exposed to higher concentrations of microcystin than in other bathing areas.

## 5. Conclusions 

Our results show that human-associated fecal contamination is associated with the toxin-producing *M. aeruginosa* at two urban beaches located in the central Lake Erie basin. This phenomenon is speculated to be linked to nutrient loading, especially nitrate. Microcystin production by toxin-producing *M. aeruginosa* may be increased when sufficient concentrations of essential nutrients such as phosphorus and nitrate are available at the beaches. Differences in the distribution of the water quality parameters may be attributed to human-associated fecal contamination, which possibly promoted cyanobacteria growth and microcystin production by nutrient loading. Therefore, it is important to identify and manage human-associated nutrient loading and fecal contamination to minimize public health risks attributable to both microcystin and potential enteric pathogens at Lake Erie beaches.
